# Study of the Crystal Structure and Hydrogen Bonding during Cold Crystallization of Poly(trimethylene 2,5-furandicarboxylate)

**DOI:** 10.1021/acs.macromol.3c02471

**Published:** 2024-02-26

**Authors:** Oscar Toledano, Oscar Gálvez, Mikel Sanz, Carlos Garcia Arcos, Esther Rebollar, Aurora Nogales, Mari Cruz García-Gutiérrez, Gonzalo Santoro, Izabela Irska, Sandra Paszkiewicz, Anna Szymczyk, Tiberio A. Ezquerra

**Affiliations:** † CICECO − Aveiro Institute of Materials, Universidade de Aveiro, Aveiro 3810-193, Portugal; ‡ Depto. Física Interdisciplinar, Universidad Nacional de Educación a Distancia (UNED), Fac. Ciencias Av. de Esparta s/n, 28232 Las Rozas de Madrid, Spain; § Instituto de Estructura de la Materia, IEM-CSIC, Serrano 121, 28006 Madrid, Spain; ∥ Instituto de Química Física Blas Cabrera, IQF-CSIC, Serrano 119, 28006 Madrid, Spain; ⊥ Department of Mechanical Engineering and Mechatronics, West Pomeranian University of Technology, Al. Piastów 19, PL 70310 Szczecin, Poland; # NANOesMAT, UNED, Unidad Asociada al CSIC por el IEM y el IQF, Av. de Esparta s/n, 28232 Las Rozas de Madrid, Spain

## Abstract

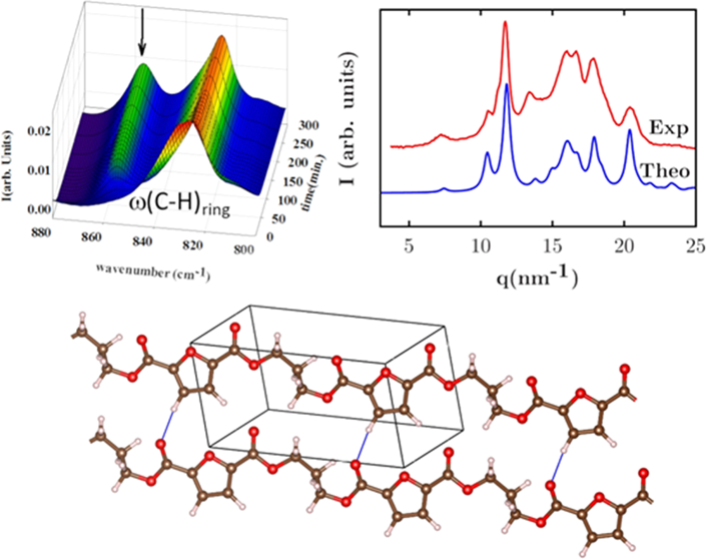

Here, we present a detailed description of the in situ isothermal crystallization of poly(trimethylene 2,5-furandicarboxylate)(PTF) as revealed by real-time Fourier transform infrared spectroscopy (FTIR) and grazing incidence wide-angle X-ray scattering (GIWAXS). From FTIR experiments, the evolution of hydrogen bonding with crystallization time can be monitored in real time, while from GIWAXS, crystal formation can be followed. Density functional theory (DFT) calculations have been used to simulate FTIR spectra for different theoretical structures, enabling a precise band assignment. In addition, based on DFT *ab initio* calculations, the influence of hydrogen bonding on the evolution with crystallization time can be understood. Moreover, from DFT calculations and comparison with both FTIR and GIWAXS experiments, a crystalline structure of poly(trimethylene 2,5-furandicarboxylate) is proposed. Our results demonstrate that hydrogen bonding is present in both the crystalline and the amorphous phases and its rearrangement can be considered as a significant driving force for crystallization of poly(alkylene 2,5-furanoate)s.

## 1 Introduction

The growing interest in lower carbon footprint polymers, as an alternative to petroleum-based ones, originates from the increasing societal need for sustainable polymer materials derived from renewable sources capable of being integrated with a circular economy concept.^1^ In recent times, fully biobased polyesters derived from furandicarboxylic acid (FDCA) have received significant attention as potential substitutes for their petrochemical-based benzene aromatic counterparts.^2,3^ Among others, poly(alkylene 2,5-furanoate)s, based on 2,5-FDCA, have aroused as promising alternatives to petroleum-based poly(alkylene terephthalate)s due to their outstanding gas barrier properties.^4,5^ In particular, poly(ethylene 2,5-furandicarboxylate) (PEF) is finding a niche in the beverage industry due to its enhanced mechanical performance and thermal stability in addition to its improved gas barrier properties in comparison to its counterpart poly(ethylene terephthalate) (PET). Moreover, the industrial production of PEF and its implementation for bottle production is close to being achieved.^6^ In order to extend this endeavor toward the future, a deep understanding of the structure–property interplay is required. In this respect, significant efforts have been recently devoted to investigating in detail the structure–property relationships of the poly(alkylene 2,5-furanoate) family,^4,5,7−10^ PEF being the most studied one. Poly(trimethylene terephthalate) (PTT) is another member of the poly(alkylene terephthalate) family with outstanding mechanical and optical properties, making it attractive for the fiber industry as well as for optoelectronic applications.^11−13^ The PTT counterpart in the poly(alkylene 2,5-furanoate)′s family is poly(trimethylene 2,5-furandicarboxylate) (PTF). In comparison to PTT, PTF exhibits a higher Young modulus, higher glass-transition temperature, lower melting point, and lower gas permeability.^5,14^ PTF has not received until now similar attention as other poly(alkylene 2,5-furanoate)s despite its potential industrial applications,^15^ and, for example, its crystalline structure has not been reported yet. Density functional theory (DFT) calculations indicate that for both PEF and PTF, an extended *anti–anti* conformation of the FDCA moiety (the two oxygen atoms of the adjacent carbonyl groups of the furan ring pointing in opposite directions) and a *gauche* arrangement of the ethylene glycol (EG) one are the most energetically favorable conformations in the amorphous state.^7−9^ On the contrary, crystallography experiments reveal that for PEF and poly(butylene 2,5-furanoate) (PBF), the syndiotactic (*syn–syn*) conformation of FDCA (the two oxygen atoms of the adjacent carbonyl groups of the furan ring pointing in the same direction) and *trans* for EG, in spite of being the least energetically favorable, prevail in the crystalline state. This is due to the additional contribution of interchain hydrogen bonding that stabilizes the crystalline structure.^7,10^ The significance of interchain and intrachain interactions in the crystallization of polylactides has been recently emphasized.^16^ Recent infrared spectroscopy experiments in PEF, PTF, and PBF suggest that, due to the statistical distribution of possible configurations, a significant fraction of *syn–syn* conformations of FDCA exist in the amorphous state.^7,9,10^ As a matter of fact, intermolecular hydrogen bonds in the amorphous phase have been proposed to play an important role in the enhanced gas barrier properties of poly(alkylene 2,5-furanoate).^5,17^ Several significant crystallization and crystalline structure studies of poly(furanoate)s have been accomplished in recent years.^18−23^ However, although hydrogen bonding seems to be the driving force for crystallization by energetic stabilization of the nonthermodynamically favorable *syn–syn* conformation of FDCA, a systematic study of the process is still missing. Vibrational spectroscopy is a powerful tool to identify at molecular-level conformations characteristic of amorphous and crystalline phases in polymers. Moreover, this type of spectroscopies, like Fourier transformed infrared (FTIR) or Raman, can be of great interest to monitor *in situ* structural modifications with potential applications for implementation in real-time industrial diagnosis.^16,24,25^

In this article, by means of FTIR and GIWAXS, we have accomplished a detailed description of the *in situ* isothermal crystallization of poly(trimethylene 2,5-furandicarboxylate) (PTF) in real time. From FTIR experiments, the evolution of hydrogen bonding with crystallization time can be monitored. DFT calculations allow us to simulate FTIR spectra for different theoretical structures. By comparing experiments and theory, a precise band assignment as well as its evolution with crystallization time can be obtained. Moreover, from DFT calculations and comparison with both FTIR and GIWAXS experiments, a proposal for the crystalline structure of poly(trimethylene 2,5-furandicarboxylate) can be achieved. Our results demonstrate that hydrogen bonding is present in both the crystalline and the amorphous phases and its rearrangement is certainly a driving force for the crystallization of poly(alkylene 2,5-furanoate)s.

## 2 Experimental Section

### 2.1 Materials

**Scheme 1:**
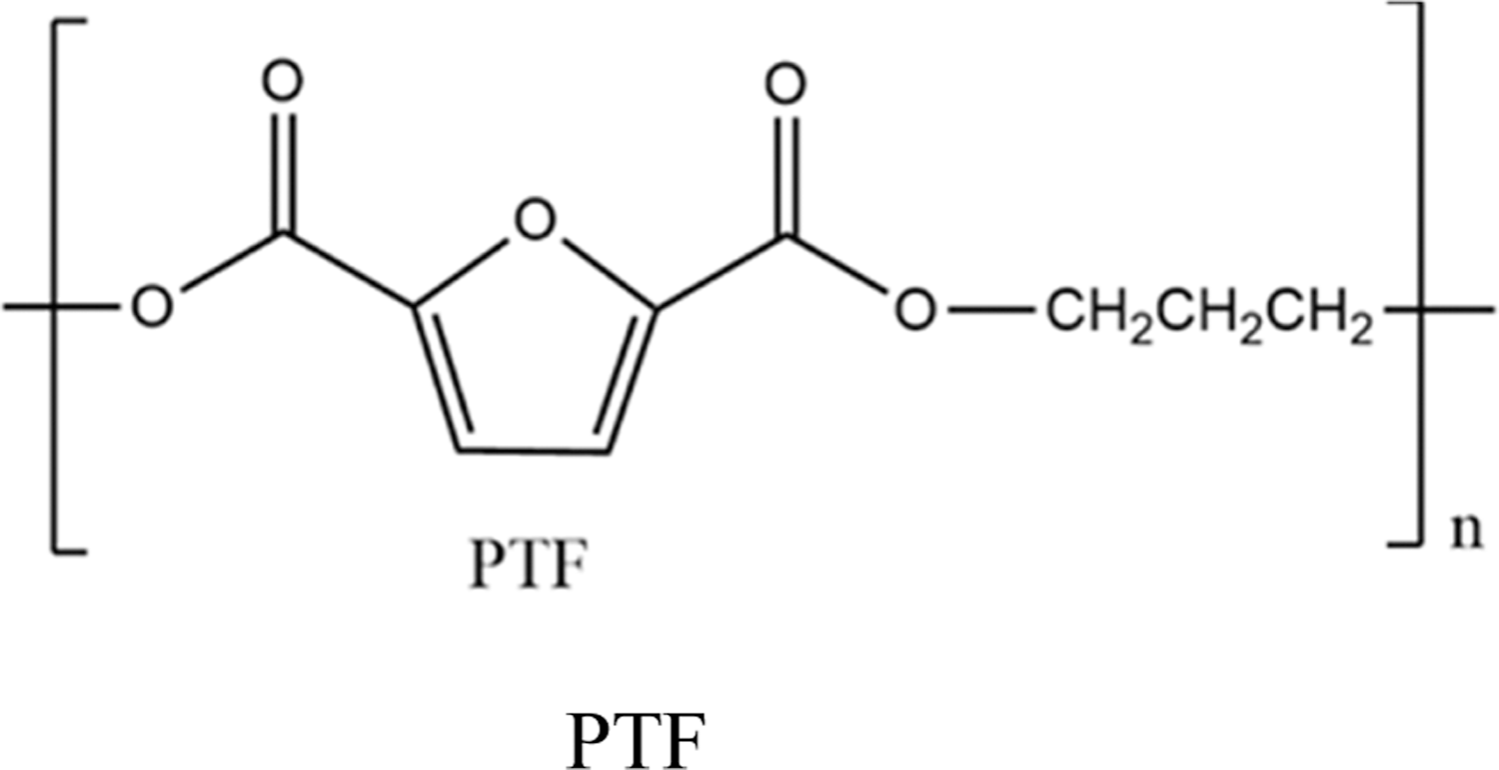
Chemical Structure of Poly(trimethylene 2,5-furandicarboxylate) (PTF)

Poly(trimethylene 2,5-furandicarboxylate) (PTF) (Scheme 1) (*M*_n_ = 34.2 × 10^3^ g mol^–1^ and *M*_w_ = 69.61 × 10^3^ g mol^–1^) was synthesized in a two-step process as previously described.^26−28^ The first step involves the transesterification reaction of dimethyl furan-2,5-dicarboxylate (DMFDCA, Matrix Fine Chemicals, Switzerland) with bio-1,3-propanediol (PDO, Susterra Propanediol, DuPont Tate & Lyle) (DMFDCA/PDO molar ratio of 1:2) in the presence of a catalyst (tetrabutyl orthotitaniate (TBT, Fluka), 0.25 wt % in relation to DMFDCA). In the second step, a polycondensation reaction takes place in the presence of the same catalyst and a thermal stabilizer (Irganox 1010, Ciba-Geigy, Switzerland, 0.5 wt % in relation to the final polymer mass).

### 2.2 Sample Preparation

Fourier transform infrared (FTIR) spectroscopy was performed in polymer thin films 900 nm thick prepared by spin-coating on FTIR transparent silicon substrates (Si FZ 25.4 mm Ø x 1 mm polished window). A 0.25 mL drop of the polymer solution in trifluoroacetic acid (Sigma-Aldrich, reagent grade ≥98%) with a concentration of 50 g/L was deposited on a circular silicon substrate. The spin-coating conditions were 2400 rpm for 120 s. Under these conditions, the spin-coated films of PTF are amorphous according to FTIR and GIWAXS measurements.^9^ The film thickness was selected in order to have an optimal FTIR absorbance without a significant band saturation effect in the whole spectral range.

### 2.3 Infrared Spectroscopy Experiments

FTIR spectra were collected in a vacuum (background pressure of 10^–7^ mbar) with a PerkinElmer Frontier spectrometer in a 4500–500 cm^–1^ range with a resolution in the wavenumber of 4 cm^–1^. Cold crystallization experiments, i.e., heating from below the glass-transition temperature, were accomplished in samples heated up from room temperature at a rate of 2.5 °C/min up to the crystallization temperature. First, FTIR spectra were registered at 25 °C. Subsequently, the temperature was increased to the crystallization temperature *T*_c_ = 80 °C by using a variable temperature cell (SPECAC). Second, FTIR spectra were recorded in real time during isothermal crystallization of the polymer. The typical acquisition time for FTIR in the investigated wavenumber range is 10 min. Background spectra were taken at both 25 °C and at the crystallization temperature for the sake of data correction.

### 2.4 Real-Time Grazing Incidence Wide-Angle X-ray Scattering Measurements during Cold Crystallization of PTF

In order to monitor by X-ray scattering the crystallization process in similar samples to those used for FTIR experiments (PTF on IR transparent silicon substrates), we used GIWAXS as described elsewhere.^29^ GIWAXS measurements were performed in the NCD-SWEET beamline at the ALBA synchrotron (Cerdanyola del Vallès, Barcelona, Spain). The X-ray beam wavelength was set at λ = 0.1 nm. An LX255-HS 2D (Rayonix) area detector placed at 0.1535 m from the sample position collected GIWAXS patterns. A standard Cr_2_O_3_ was used to calibrate the sample-to-detector distance, detector tilts, and reciprocal space. The scattered intensity was corrected for beam polarization and background. GIWAXS patterns were acquired with an incident angle of 0.2°. The scattered intensity was azimuthally integrated, and one-dimensional diffractograms were obtained, representing the scattered intensity versus the modulus of the scattering vector *q*, where *q* = 4π/λ(sin θ) and 2θ is the scattering angle. A modified Linkam hot stage was used for temperature control in the isothermal crystallization experiments. Cold crystallization experiments were accomplished in samples heated up from room temperature at 30 °C/min up to the crystallization temperature. GIWAXS patterns were acquired every 60 s with a 1 s acquisition time. The sample was displaced 100 μm perpendicular to the X-ray beam to avoid radiation damage. The temperature for both GIWAXS and FTIR was selected to provide enough time to enable comparison with FTIR data.

### 2.5 *Ab Initio* Calculation

Quantum mechanical calculations were performed in the framework of the density functional theory (DFT). Simulations were performed using the SIESTA package,^30^ which employs an atomic orbital basis set and pseudopotentials to reproduce the interaction with the core electrons. To take into account the intermolecular interactions and the exchange–correlation energy, the vdW-DF-cx^31^ functional was selected, which includes dispersive corrections that are significant in these types of systems. In this work, we have employed a double-ζ polarized (DZP) basis set, truncated with an energy shift of 40 meV. Standard Troullier–Martins norm conserving pseudopotentials taken from abinit pseudopotential database were employed.^32^ The real spatial grid is set to 1200 Ry (which leads to a spatial resolution of approximately 0.045 Å), while the reciprocal space has a resolution equivalent to performing the calculation in a supercell with always more than 20 Å in each direction with a single Γ-point. The positions of the atoms and the unit cell parameters were relaxed until the forces and stresses were lower than 0.001 eV/Å and 0.1 GPa, respectively. The frozen phonon method was used to obtain the vibrational frequencies. A displacement of 0.04 Bohr was allowed to compute the dynamical matrix elements. To evaluate the infrared activities of the vibrational modes, the Born charges were computed as implemented in the SIESTA code. In order to represent the predicted infrared spectra, the peaks were broadened by Lorentzian functions with a half-width at half-maximum (HWHM) of 8 cm^–1^. As pointed out in a previous work,^9^ the vibrational frequencies of the carbonyl group are systematically underestimated by the vdw-DF functionals. Thus, a factor of 1.036 was applied in the frequency range of this normal mode.^33^ Theoretical powder diffraction patterns were calculated using VESTA software,^34^ and the Bragg diffraction peaks were broadened by Lorentzian functions with a HWHM of 0.8°.

## 3 Results and Discussion

### 3.1 Real-Time Crystallization of PTF as Revealed by GIWAXS Experiments

**Figure 1:**
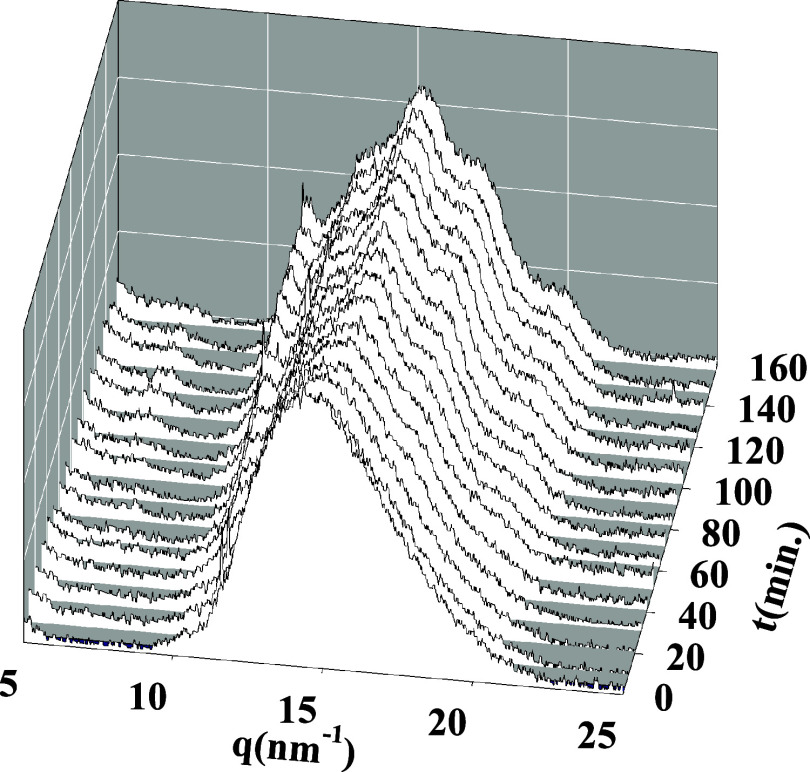
Isothermal cold crystallization at 80 °C of the initially amorphous PTF followed by GIWAXS. Intensities are represented as a function of scattering vector *q* and as a function of the crystallization time in minutes (*t*(min)).

Isothermal experiments during *in situ* cold crystallization of PTF were performed by GIWAXS experiments using synchrotron radiation. Special care was taken to ensure that samples were amorphous at the beginning of the crystallization experiment. Figure 1 illustrates the evolution of the GIWAXS patterns with crystallization time for a crystallization temperature of 80 °C. Higher or lower crystallization temperatures provide either too slow or too fast crystallization processes as to allow efficient FTIR data collection in the broad wavenumber range investigated.

**Figure 2:**
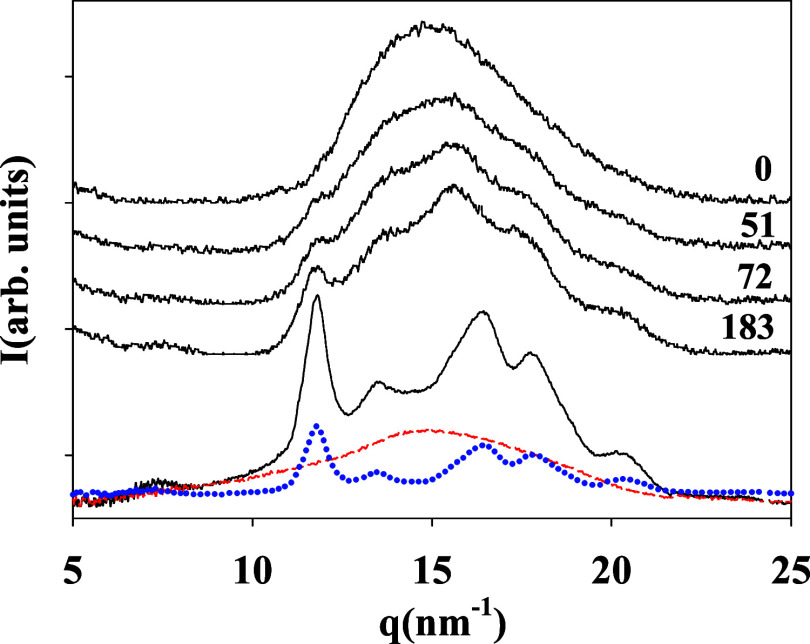
Diffractograms of the *in situ* isothermal cold crystallization of PTF (*T*_c_ = 80 °C). The labels on the right indicate the crystallization time in minutes. The bottom diffractogram (continuous line) corresponds to an *ex situ* crystallized PTF sample (*T*_c_ = 160 °C, *t* = 6 h). The deconvolution procedure to estimate the crystallinity is illustrated by showing the amorphous halo contribution (red dashed line) and by the crystal phase contribution (blue dotted line).

For the initial times, the patterns are characterized by a broad maximum, which is typical of amorphous materials, confirming the initial amorphous nature of the sample. As temperature increases, Bragg peaks appear superimposed on the amorphous halo due to the crystallization process. This is a standard behavior occurring during the crystallization of polymers.^35,36^Figure 2 shows selected GIWAXS patterns along the crystallization experiment for some characteristic times.

**Figure 3:**
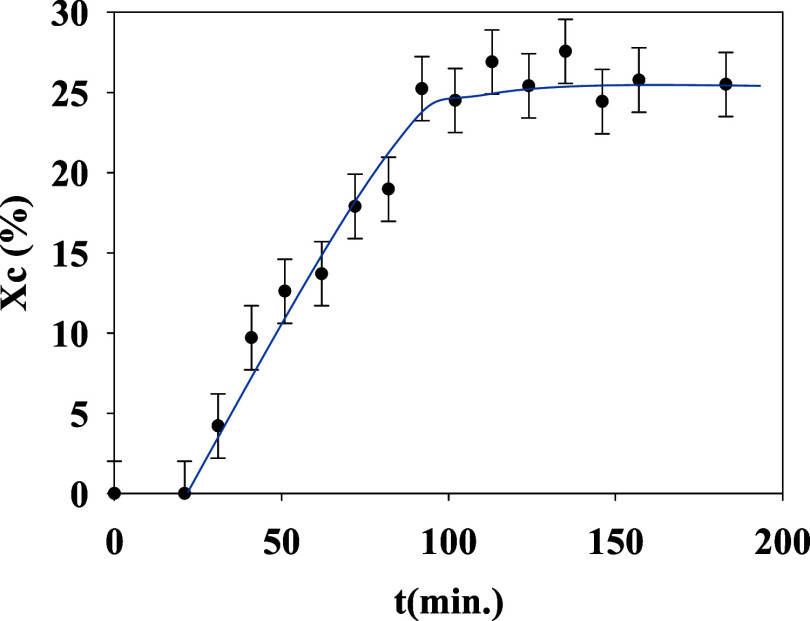
Crystallinity (*X*_c_) as a function of crystallization time, in minutes, for isothermal cold crystallization of PTF at 80 °C. Continuous line is a guide to the eye.

The diffractograms are in accordance with those previously published.^19^ For the sake of discussion, a PTF sample crystallized *ex situ* was prepared, aiming to obtain higher crystallinity. In this case, a PTF film 200 μm thick was crystallized at *T*_c_ = 160 °C for 6 h in a Linkam hot stage. The corresponding diffractogram is included in Figure 2. The degree of crystallinity, *X*_c_, can be obtained from the X-ray diffractograms.^37,38^ The total scattering pattern was considered a linear combination of the crystalline (*X*_crystal_) and amorphous contributions (*X*_a_): *I*(*q*,*t*)= *X*_crystal_*I*_c_(*q*,*t*) + [1 – *X*_crystal_]I_a_(*q*,*t*), where *X*_crystal_ is the fraction of the crystalline phase, *I*_c_ (*q*,*t*) is the intensity from the Bragg peaks, and *I*_a_(*q*,*t*) is the intensity from the amorphous halo. Thus, crystallinity can be calculated from the ratio between the area below the crystalline peaks, *A*_c_, to the total scattered area, *A*_c_ + *A*_a_, by *X*_c_ = *A*_c_/(*A*_c_ + *A*_a_). The contribution of the amorphous halo was taken considering the initial pattern (crystallization time *t*_c_ = 0). An illustration of the procedure is shown in Figure 2. The evolution of the crystallinity with time is shown in Figure 3.

After an initial induction time, a primary crystallization process occurs where crystallinity values increase dramatically. Afterward, a secondary process takes over where crystallinity increases at a significantly slower rate. This behavior is quite common for isothermal cold crystallization of polymers and qualitatively similar to that observed for PTT.^39^ As expected, a higher value of *X*_c_ = 46% is obtained for the *ex situ* crystallized sample at a higher temperature for longer times.

### 3.2 FTIR of PTF

**Figure 4:**
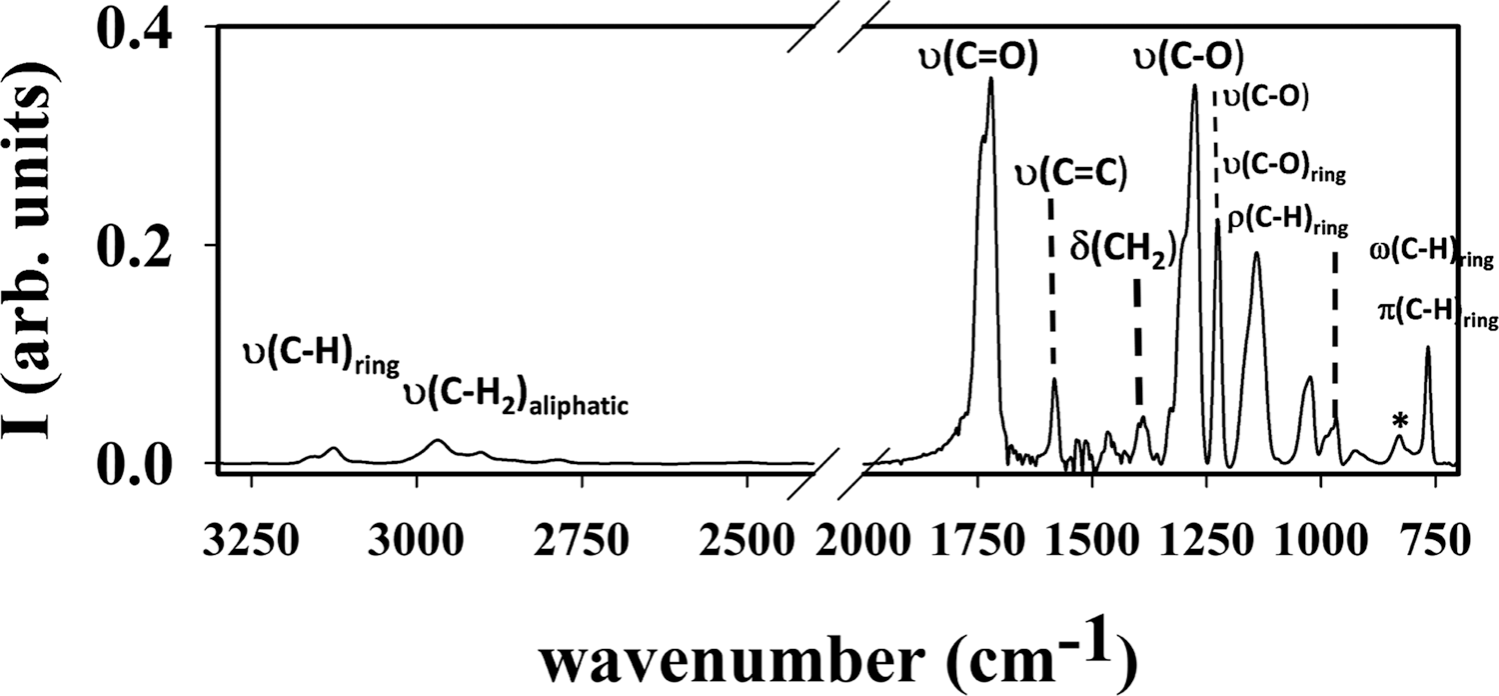
FTIR absorption spectrum of PTF at *T* = 25 °C. The main chemical groups associated with the absorption features are labeled: ν stretching, ρ bending in plane, ω wagging out of plane, and π out-of-plane deformation modes.

Figure 4 shows the FTIR absorption spectra of PTF at 25 °C. The FTIR spectrum of the PTF corresponds to a fully amorphous sample, as proven by GIWAXS and in accordance with previous studies.^7,9,40^

The main normal modes associated with the absorption bands of amorphous PTF have been indicated in Figure 4. Table 1 collects all of the different bands and the assignment of the vibration normal modes proposed. The assignment was carried out based on our *ab initio* calculations and previous studies.^7,9,40^

**Table 1:** Wavenumber (cm^–1^) of the Absorption Bands for PTF (Amorphous and Semicrystalline Samples) and Assignment of the Normal Modes of Vibration: ν Stretching, ρ Bending in Plane, ω Wagging out of Plane, and π out-of-Plane Deformation Modes^a^

amorphous			semicrystalline		
wavenumber	vibrational mode	*I*_rel_	wavenumber	vibrational mode	*I*_rel_
3155, 3127	ν_sy_(C–H)_ring_, ν_asy_(C–H)_ring_	0.02; 0.04	3149, 3118	ν_sy_(C–H)_ring_, ν_asy_(C–H)_ring_	0.04; 0.07
2968, 2903	ν_asy_(C–H_2_), ν_sy_(C–H_2_)	0.06; 0.03	2968, 2905	ν_asy_(C–H_2_), ν_sy_(C–H_2_)	0.06; 0.03
1738, 1721	ν(C=O)_syn_, ν(CO)	0.85; 1.00	1737, 1730	ν(C=O)_syn_, ν(CO)	0.92; 0.98
1583	ν(C=C)	0.22	1581, 1573	ν(C=C)	0.20; 0.20
1466	δ(CH_2_)	0.08	1468	δ(CH_2_)	0.14
1276	ν(C–O)	0.98	1276	ν(C–O)	0.93
1226	ρ(C–H)_ring_, ν(C–O)_ring_, ν(C–O)	0.63	1306^sh^, 1226	ρ(C–H)_ring_, ν(C–O)_ring_, ν(C–O)	0.54
1141	ρ(C–H)_ring_, ν(C–O)_ring_, ν(C–O)	0.55	1153	ρ(C–H)_ring_, ν(C–O)_ring_, ν(C–O)	0.58
1024	ρ(C–H)_ring_, ν(C–O)_ring_, ν(C–O)	0.22	1036	ρ(C–H)_ring_, ν(C–O)_ring_, ν(C–O)	0.21
967	ν(C–O)_ring_, ν(C–O)	0.12	982^sh^, 967	ν(C–O)_ring_, ν(C–O)	0.17
926	ν(C–O)_ring_, ν(C–O)	0.03	928	ν(C–O)_ring_, ν(C–O)	0.06
829	ω(C–H)_ring_	0.07	856, 825	ω(C–H)_ring_	0.03; 0.05
766	ω(C–H)_ring_, π(C–O–C)_ring_	0.30	773, 766	ω(C–H)_ring_, π(C–O–C)_ring_	0.23; 0.23

a *I*_rel_ is the relative intensity of the bands. ^sh^ is for shoulder.

### 3.3 Real-Time Crystallization of PTF as Revealed by FTIR Experiments

**Figure 5:**
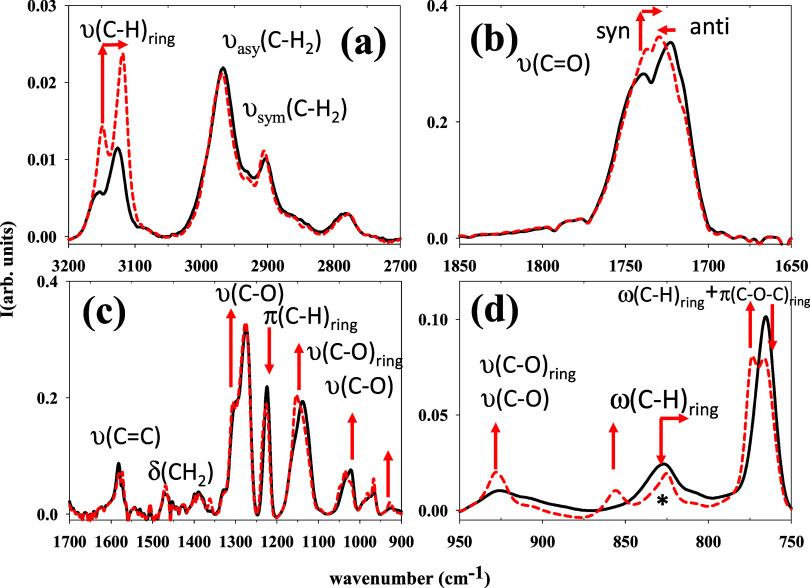
FTIR absorption spectra of PTF in different spectral regions during isothermal cold crystallization at *T* = 80 °C for (continuous black line) initially amorphous and (dashed red line) the final semicrystalline PTF. From Figure 5a–d, the main chemical groups associated with the absorption features are labeled. The arrows indicate growth, decrease, or displacement with time of significant bands during crystallization. The asterisk marks the band around 829 cm^–1^ (see the main text).

Isothermal cold crystallization of PTF was monitored *in situ* by FTIR. Special care was taken to ensure that the samples were amorphous at the beginning of the crystallization experiment. Figure 5 shows the spectra of PTF at *T* = 80 °C for the initial crystallization time and after 280 min at 80 °C. The initial spectrum is essentially similar to that at 25 °C, supporting the amorphous character of the sample at the beginning of the isothermal crystallization. For the sake of clarity and considering the difference in intensities of the various bands, the spectra have been divided into characteristic spectral ranges.

As illustrated in Figure 5, the main variations of the infrared bands are associated with normal modes that involve the hydrogen atoms of the furan ring (C–H)_ring_ or the C=O bonds. This suggests that strong interchain hydrogen bonds are established among these groups during the crystallization. In particular, for the absorption bands around 3200–3100 cm^–1^ assigned to the ν(C–H)_ring_ (Figure 5a), one observes an intensity increase accompanied by a red shift (reduction of wavenumber) during the crystallization process. This behavior is expected if strong interchain hydrogen bonds among hydrogen atoms of the furan ring and C=O groups are formed. In this case, (C–H)_ring_ bonds should become more polarized and reduce their strength. It is also observed that the bands around 3000–2900 cm^–1^, assigned to the stretching mode of the C–H bonds of the aliphatic chain, remain practically unaltered during crystallization. This indicates that the chemical environment of these bonds is not significantly modified during crystallization. Notable modifications occur in the spectral region where the bands associated with the C=O stretching band appear (Figure 5b). Thus, the peak ascribed to *syn* conformation increases during the crystallization process. This effect suggests that a fraction of the C=O bonds, originally in *anti* conformation in the amorphous phase, transform into a *syn* conformation during the crystallization. It is worth mentioning that *syn* conformations are those involved in interchain hydrogen bonding.^7,9^ Major changes are also observed in the infrared region from 1350 to 950 cm^–1^ (Figure 5c), in which different shoulders or peaks emerge during the crystallization. The most drastic changes involve bands with a larger participation of ρ(C–H)_ring_ and v(C–O)_ring_ normal modes. These observations are compatible with the formation of strong interchain hydrogen bonds involving these groups. Special attention should be paid to the spectral region assigned to the ω(C–H)_ring_ and π(C–O–C)_ring_ normal modes (Figure 5d). Our calculations predict that the band at 829 cm^–1^ (marked with an asterisk in Figures 4 and 5) is only observed if interchain hydrogen bonds involving the (C–H)_ring_ bond are present. This will be discussed in the next paragraph. Curiously enough, this band is already observed at room temperature (Figure 1), as well as at 80 °C at *t* = 0, suggesting the presence of a fraction of hydrogen bonds in the amorphous phase as previously proposed.^7,9^ Moreover, when crystallization proceeds, a new band around 856 cm^–1^ arises in this spectral region. This indicates that (C–H)_ring_ bond rearrangements are involved in forming the crystalline phase. We have calculated that the integral area of both peaks (centered at 825 and 856 cm^–1^) in the final stage of crystallization is similar to that of 829 cm^–1^ in the initial amorphous state. This fact could indicate that during crystallization, a rearrangement of interchain interactions rather than the formation of new ones takes place. This rearrangement during crystallization could involve a strengthening of the interchain hydrogen bonds as well as the formation of new ones.

**Figure 6:**
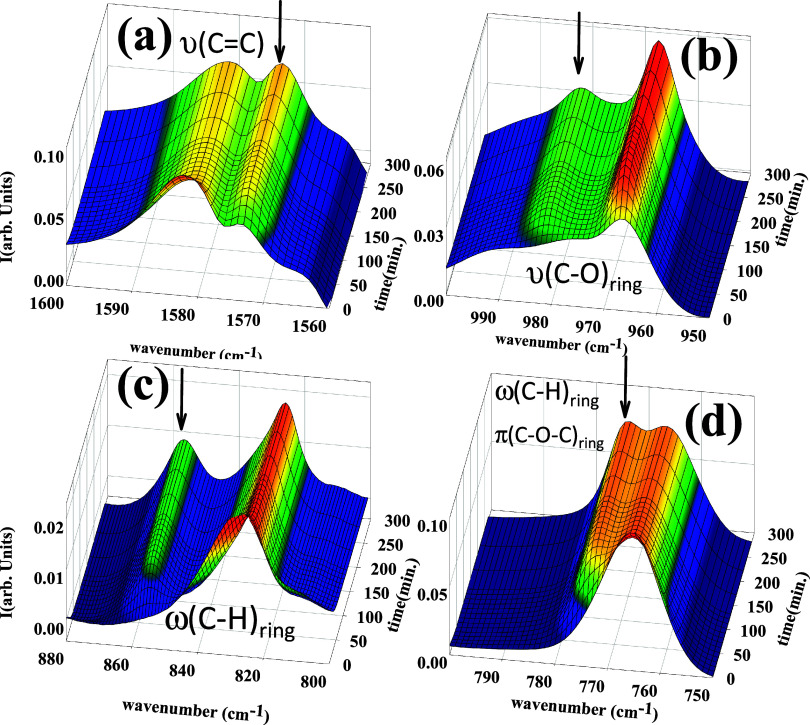
FTIR absorption spectra as a function of crystallization times of selected crystallinity-dependent bands of PTF during isotherm crystallization. Panels (a–d) are for the infrared spectral regions centered around 1573, 982, 856, and 773 cm^–1^ (marked by arrows), respectively.

**Figure 7:**
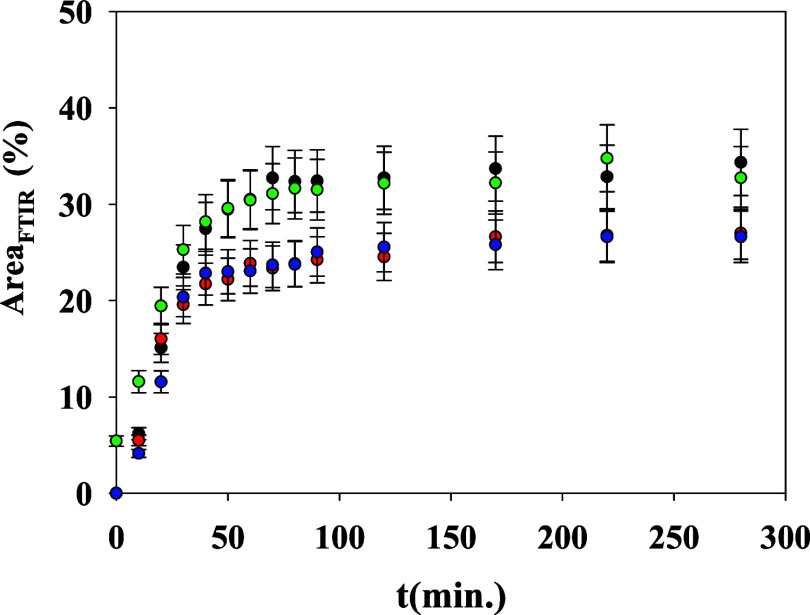
Ratio of the newly emerged band to the total infrared band during the crystallization process for selected bands of PTF. (Black circle) 856 cm ^–1^, (red circle) 774 cm^–1^, (green circle) 1573 cm^–1^, and (blue circle) 967 + 982 cm^–1^.

Some infrared bands present significant variations during the crystallization process. An example of those crystallinity-dependent bands is shown in Figure 6. In these cases, a deconvolution of the different contributions to the infrared absorption has been accomplished. A precise description of the procedure is included in the Supporting Information (section A1). From this analysis, a percentage of the band area increment in relation to the total area of the spectral region considered can be estimated. The dependence with crystallization time on the percentage of increment of the total area for the crystallinity-dependent bands is presented in Figure 7. The absence of induction time is to be noticed in this case as compared to data in Figure 3. While FTIR molecular vibrations are probed by GIWAXS, we probe the presence of crystals by the appearance of Bragg peaks. The results suggest that the molecular vibration of particular groups may start varying before the crystal appears because of the rearrangement of the melt previous to the onset of crystal appearance. Simultaneous FTIR and GIWAXS experiments would be needed to further elucidate this point. The increment percentage of 30% of the total area of the peak emerging during the crystallization is similar for all of the bands analyzed, and it can be associated with the formation of strong interchain hydrogen bonds. The main variations for all of the bands analyzed occur during the first 60 min at 80 °C, and only small changes are observed after this time. This agrees with the results observed from GIWAXS in which the primary crystallization process also takes place in the first 60 min (see Figure 3).

### 3.4 Quantum Mechanical Molecular Simulations

#### 3.4.1 Crystalline Structure of PTF

**Figure 8:**
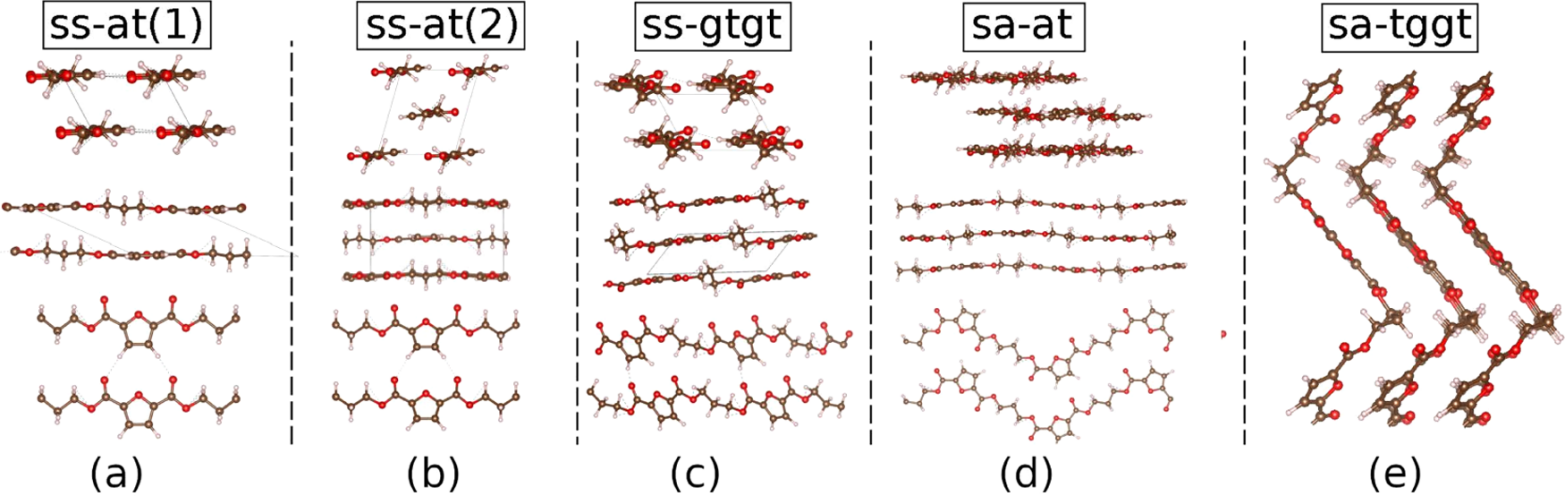
Schematic views of the evaluated model crystalline structures for PTF. Structures are labeled depending on the presence of conformations *syn–syn* (*ss*) and *syn–anti* (*sa*) of the FDCA moiety and *all-trans* (*at*), *gauche–trans–gauche–trans* (*gtgt*), and *trans–gauche–gauche–trans* (*tggt*) for the 1,3-propanediol (PDO). The schemes are labeled as structures with (a) ss–at (1), (b) ss–at (2), (c) ss–gtgt, (d) sa–at, and (e) sa–tggt.

In order to gain a quantitative insight into the changes occurring during crystallization of PTF, we have performed *ab initio* quantum mechanical calculations. As the crystalline structure of PTF has not been reported, we have evaluated the thermodynamic stability of several possible crystalline structures. We have probed several molecular crystalline configurations based on those proposed for similar polymers like PEF,^20,41^ PBF,^42^ or PTT.^43^ First, we have compared their relative energy stability to discriminate the most favorable crystalline structure. Next, the powder diffraction patterns and the infrared spectra of the different proposed structures have been computed as described in the Experimental Section. Finally, a comparison with the experiments has been accomplished. As pointed out in previous studies^9^ and corroborated by our own calculations (see Figure SF3 in the Supporting Information), the *anti–anti* configuration of the 2,5-furandicarboxylic acid (FDCA) moiety of the PTF structure is the most stable in the absence of intermolecular interactions. However, in condensed phases, the *syn* conformations yield lower energy than the *anti* configurations due to the formation of intermolecular hydrogen bonds between the carbonyl groups and the hydrogen atoms of the furan ring of the neighboring polymer chains (more details are given in Section A2.1 of the SI). The formation of these strong interchain interactions explains that crystalline structures of other poly(2,5-furanoate)s like PEF or PBF exhibit FDCA *syn–syn* or *syn–anti* conformations. Figure 8 shows five probable crystalline structures selected to be candidates for that of PTF. A detailed description of the methodology followed in selecting these initial unit cells on the basis of the different conformers can be found in the Supporting Information.

Structures (a) *ss–at*, (b) *ss–at*, and (d) *sa–a*t in Figure 8 are analogous to the α, α′, and β crystalline structures of PEF, respectively.^20,41^ Structure (c) *ss-gtgt* is proposed in concordance with the crystalline configuration suggested for the PBF.^42^ Finally, configuration (e) *sa–tggt* is analogous to the crystalline structure found for PTT.^43^ These configurations are labeled according to the orientation of both the carbonyl groups of the FDCA motif, which can occur in *syn–anti* (*sa*), or *syn–syn* (*ss*), and to the conformation of 1,3-propanediol (PDO), which could be found in these structures in *all-trans* (*at*), *gauche–trans–gauche–trans* (*gtgt*), or *trans–gauche–gauche–trans* (*tggt*) configuration. It is worth mentioning that both configurations (a) and (b) have an *ss–at* structure. However, configuration (a) presents one polymeric chain per unit cell, while configuration (b) allocates two polymeric chains with opposite orientations in the unit cell. Table 2 collects the energy calculations for the different structures of Figure 8 in addition to the calculated lattice parameters.

**Table 2:** Energy per Monomer (eV), Lattice Parameters (Angstroms (Å)), Volume per Monomer, and Density of the Different Models Shown in Figure 8^a^

	Δ*E*/mon (eV)	*a*	*b*	*c*	α	β	γ	Vol/mon (Å^3^)	density (g/cm^3^)
(a) ss–at (1)	0.09	5.86	8.28	11.82	26.6	90.3	102.8	224.5	1.450
(b) ss–at (2)	0.09	5.90	6.68	11.82	90.0	90.0	74.1	224.4	1.450
(c) ss–gtgt	0.00	5.88	4.60	11.00	122.4	105.2	103.9	214.4	1.518
(c) ss–gtgt–fit	0.14	6.12	4.61	11.07	119.0	117.0	86.5	238.5	1.366
(d) sa–at	0.24	6.71	8.22	21.08	90.0	90.00	127.6	230.7	1.410
(e) sa–tggt	0.07	6.53	4.52	16.73	90.0	85.9	110.3	231.0	1.409

a In the case of structure (c), the values used for the fitting to the X-ray diffraction pattern have been included (*ss–gtgt–fit*).

**Figure 9:**
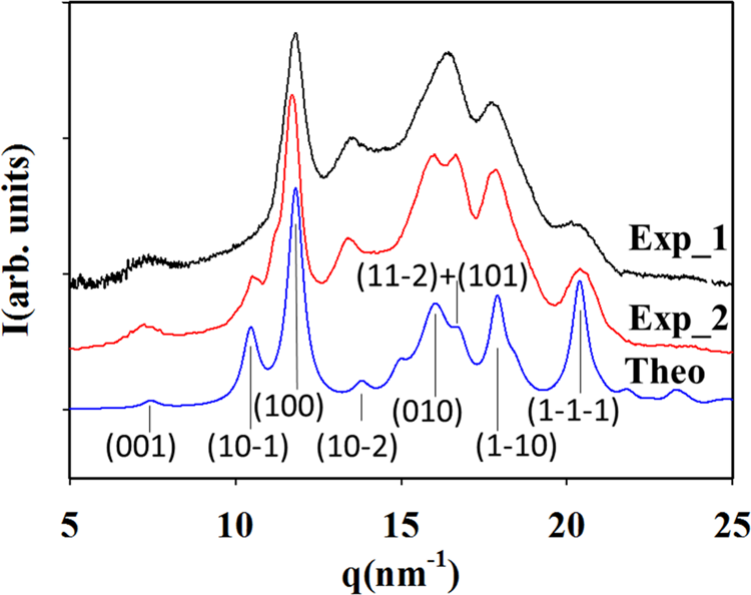
Experimental and theoretical X-ray diffraction patterns of PTF as a function of the modulus of the scattering vector *q*. In blue, the theoretical pattern of the (c) fitted crystalline structure. In black, that obtained at *T*_c_ = 160 °C for *t* = 6 h. In red, that extracted from the literature^19^ corresponding to a PTF melt crystallized following the self-nucleation procedure. Miller indices are presented at the bottom.

The modeling predicts configuration (c), with *syn–syn* conformation, to be the most energetically stable. However, it is to be noted that the energy differences per monomer among the different structures are very small as compared to the thermal energy at room temperature, considering that the monomer unit is composed of 22 atoms and *k*_b_*T* ∼ 0.026 eV at 25 °C. Therefore, even if model (c) presents the lowest energy, the other structures cannot be discarded as possible candidates for the crystalline phase. Figure 8 illustrates that configuration (c) presents strong hydrogen bonds between one carbonyl group and the hydrogen of the furan ring of the neighboring molecule with an intermolecular distance of 2.06 Å. However, the interaction of the other carbonyl group with another hydrogen atom of the neighboring molecule is much weaker as the distance between both groups is larger. The higher stability of this structure as compared to that of (a) and (b) configurations, where more symmetrical and intense interchain hydrogen bonds are established, is due to the higher stability of the “tgtg” configuration when compared with the “all-trans”. As the crystalline structure of PTF has not been reported, we have compared the predicted X-ray powder diffraction patterns of the simulated crystalline structures with the experimental ones. In order to approach experimental conditions, the predicted unit cell lengths and angles were allowed to vary a maximum of 0.5 Å and 20°, respectively, from those obtained after a complete energy minimization. This is necessary because thermal expansion of the unit cell at room temperature is not contemplated by the *ab initio* calculations. After cell parameter modification, a further relaxation of the atomic positions with the altered unit cell was performed, ensuring that the resulting final configuration is energetically stable. Figure 9 shows the simulated diffraction patterns for structure (c) described in Figure 8. In addition, we have included in Figure 9 an experimental pattern of a well cold-crystallized PTF sample (*T*_c_ = 160 °C, *t* = 6 h; Figure 2) and another diffractogram from the literature^19^ corresponding to a PTF crystallized from the melt following the self-nucleation procedure.^44^ The simulated diffractograms of structures (a), (b), (d), and (e) lead to diffraction patterns that do not match the experimental ones (see Figure SF6 in the SI).

The unit cell parameters and the energy calculation of the expanded structure (c) (*ss–gtgt–fit*) are gathered in Table 2. As an additional SI file, we have included a *cif* file with the atom positions corresponding to the crystalline structure (c_fit_). The expanded (c) structure is only 0.14 eV less stable than the fully relaxed unit cell. As mentioned above, this small distortion from the entirely minimized structure could be explained by the effect of temperature. As DFT simulations are performed without considering the temperature contribution then, a cell expansion should be expected at room temperature. This effect can be significant in systems where the unit cell dimensions are determined by intermolecular interactions. In fact, the measured density of the semicrystalline PTF with a degree of crystallinity of *X*_c_ = 31% has been reported^14^ to be ρ = 1.377 g/cm^3^, rendering a crystalline density of ρ = 1.426 g/cm^3^. Our predicted value for the fitted structure ρ_c-fit_ = 1.366 g/cm^3^ is slightly lower than this but closer than that found for the originally predicted (c) structure, ρ = 1.518 g/cm^3^. The good agreement of the X-ray diffraction pattern along with the higher stability of the (c) structure suggests that the crystalline structure of PTF is compatible with the configuration (c_fit_).

#### 3.4.2 Simulated Infrared Spectrum of PTF

As described above, the infrared spectrum of PTF was recorded during *in situ* crystallization (Figure 6). Before continuing the discussion, let us remember that at the end of the crystallization process, both an amorphous and a crystalline phase coexist with a crystallinity degree of around 25%, as revealed by the GIWAXS measurements (Figure 3). Focusing on the FTIR spectra before and after the crystallization process (Figure 5), significant changes in several vibrational bands are obtained. These changes can help us unveil how the different parts of the polymeric chain interact in the amorphous state and when and how the crystalline phase is being formed. In order to evaluate the stability of the different conformers of the PTF monomer, we have performed DFT calculations on three different types of systems: first, an isolated PTF model monomer, composed of two PDOs and one FDCA units, PDO_2_FDCA_1_. Second, a 1D model PTF chain consisting of an isolated thread with periodic boundary conditions in the direction of the thread. Finally, a bulk crystalline structure with periodic boundary conditions in all directions. Here, we will present the results obtained for the model molecular monomer and for crystalline structure. The calculations for the 1D model PTF chain are included in the SI part.

**Figure 10:**
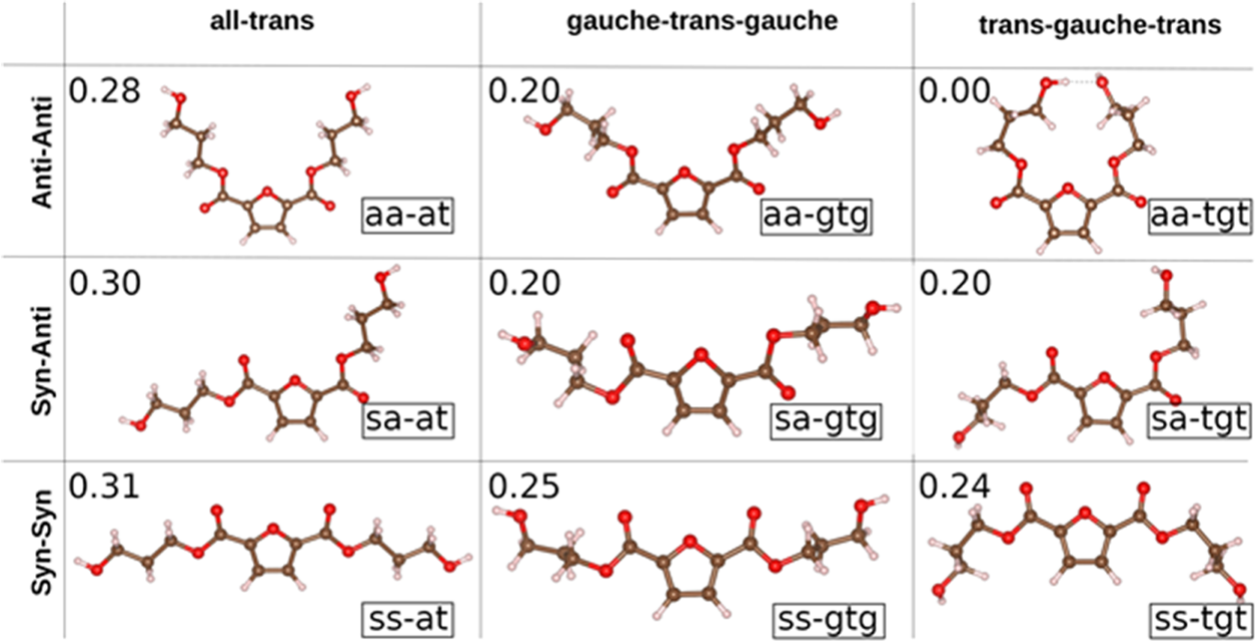
Nine investigated molecular conformations of PDO–FDCA. Their relative energy (eV) is displayed in the left-top corner of each configuration, taking as reference the energy of the most stable configuration found, which is the aa–tgt.

First, we have evaluated the stability of different single conformers, i.e., without intermolecular interactions, formed by PDO and FDCA, shown in Figure 10.

**Figure 11:**
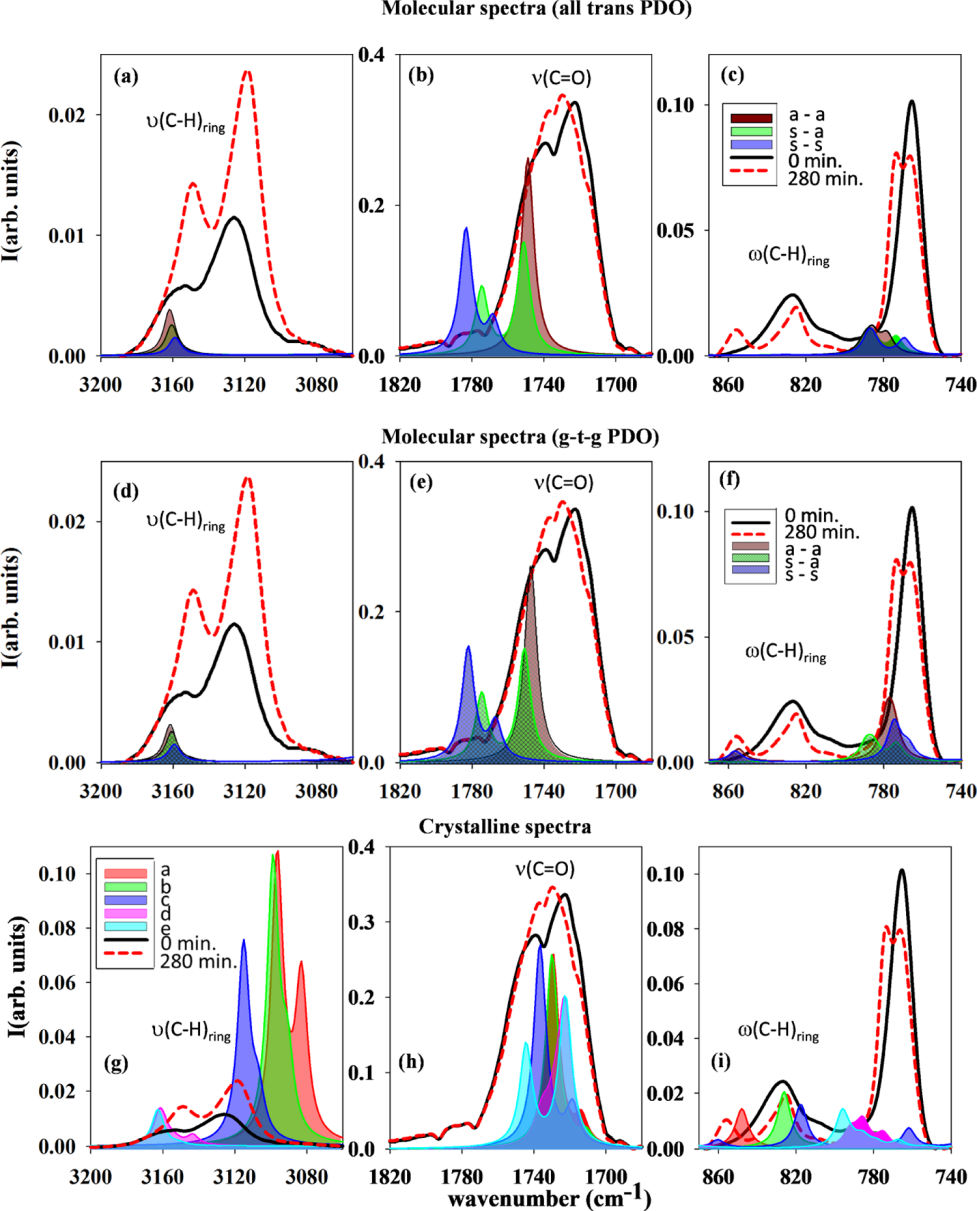
FTIR absorption spectra for the molecular configurations (top and middle) and for the crystalline models (bottom) for three representative spectral ranges. For the molecular spectra, the labels refer to a–a, s–a, and s–s for *anti–anti*, *syn–anti*, and *syn–syn* conformations of the FDCA group, respectively. First row (a–c): all cases for the all-trans for the PDO part. Second row (d–f): g–t–g (gauche–trans–gauche) for the PDO part. For the crystalline spectra (g–i), the labels read a, b, c, d, and e for the theoretical crystalline structures shown in Figure 8. In all cases, the theoretical spectra have been compared with the experimental ones: 0 min (initial FTIR spectrum at 80 °C) and 280 min (FTIR spectrum after 280 min at 80 °C).

In Figure 10, we can see that the *anti*–*anti* configurations (first row in Figure 10) lead to the most stable structures, the *syn*–*syn* (third row) being the least stable ones. It can also be noticed that the all-trans configurations are less stable than those that include gauche defects. The simulated FTIR spectra for these isolated conformers were compared to those obtained for the crystalline models (Figure 8). For the sake of clarity, we present in Figure 11 the spectra for the *syn–syn* (ss), *syn–anti* (sa), and *anti–anti* (aa) conformations of the FDCA group with *all-trans* for the PDO part in the first row (Figure 11a–c) and those corresponding to the g–t–g PDO conformations in the second row (Figure 11d–11f). The spectra for the other conformations are shown in the SI (Figure SF3). The spectra for the crystalline structures are shown in Figure 11g–11i. We will focus on three regions of the absorption spectra: that corresponding to the C–H stretching of the furan ring, ν(C–H)_ring_, which appears at ∼3150 cm^–1^; the one corresponding to the stretching of the carbonyl group C=O, ν(C=O), occurring at ∼1720 cm^–1^; and that in the range of 900–750 cm^–1^ associated with the C–H wagging of the furan ring, ω(C–H)_ring_. These three spectral ranges are included in Figure 11, where both experimental amorphous and crystalline spectra are compared with the calculated spectra for six different molecular conformations out of the nine studied (Figure 10) and with those of the crystalline structures described in Figure 8. The spectral region of the ν(C–H)_ring_ mode (Figure 11a or 11d or 11g) shows two differentiated absorption bands, separated by Δν ∼ 30 cm^–1^, that are present before and after crystallization. However, the calculated spectra in this spectral range for the isolated molecular conformers exhibit a single and very similar vibrational band. It is worth considering that for the calculations performed in the molecular conformers, the interchain hydrogen bonds between the hydrogen atoms of the furan ring and the carbonyl groups of neighboring molecules are not considered. Thus, the appearance of a bimodal band in this spectral range for the crystalline model can be attributed to interchain hydrogen bonding. In fact, the ν(C–H)_ring_ band for the crystalline models with *syn–syn* configurations (Figure. 8), which indeed present strong intermolecular hydrogen bond, is shifted toward lower wavenumbers (red shift) in comparison to either those for the molecular models or those for the crystalline models with *syn–anti* conformations. Moreover, this red shift is larger in the case of structures (a) and (b), where two symmetrical hydrogen bonds are produced, than in structure (c), where only one of the hydrogen atoms participates in the interchain interactions. It is to be noticed that, for the *syn–anti* crystalline models, (d) and (e), exhibiting weaker hydrogen bonding, the predicted bands almost coincide with those of the molecular conformers.

Accordingly, the experimental occurrence of a bimodal band for the ν(C–H)_ring_ mode in the amorphous PTF sample can be attributed to the presence of interchain hydrogen bonds as previously suggested.^9,17^ It is known that the symmetrical and asymmetrical normal modes of ν(C–H)_ring_ could cause a bimodal character of this band. However, according to our calculations, this splitting is only of about 11 cm^–1^, which do not match with the observed splitting of about 28 cm^–1^. Furthermore, the asymmetric vibrational mode occurs at lower frequencies and is always predicted to be much less intense (more than 10 times for all of the molecular conformations) than the symmetrical mode, which is also inconsistent with the experimental observations. It is to be considered that the band at ∼3160 cm^–1^ could be assigned to the overtone of the asymmetric C=C stretching. However, the strong increment of the intensity of this band during the crystallization (which is almost twice as much as that of the amorphous phase; see Table 1) and the negligible intensity change undergone in the case of the fundamental normal mode ν(C=C), at 1583 cm^–1^, points against this possible assignment.

Very striking is the situation for the 830–820 cm^–1^ spectral region where the ω(C–H)_ring_ is active (Figure 11c,f). The simulations shown in Figure 11 show that the molecular models (without hydrogen bonding) for anti–anti, syn–anti, and syn–syn conformations of the FDCA group exhibit no bands in the 829 cm^–1^ spectral region neither for all-trans nor for gauche–trans–gauche PDO conformations. However, the simulated spectra for the crystalline models exhibit the presence of absorption bands in the 850–820 cm^–1^ spectral range, further emphasizing the occurrence of hydrogen bonding in the amorphous phase. In fact, for the case of crystalline structures (structure “a”, all-trans, and “c”, gtgt), in which strong intermolecular hydrogen bonds occur, the predicted bands for ω(C–H)_ring_ are 848 and 818 cm^–1^, respectively. This is consistent with the bands of the semicrystalline sample at 825 and 856 cm^–1^ and even with the presence of the band at 829 cm^–1^ in the amorphous phase. It is true that an additional band appears around 856 cm^–1^ only in the molecular spectra of anti–anti and syn–syn conformations of the FDCA for gauche–trans–gauche PDO conformations (see Figure 11f). However, experimentally, this band is not observed in the amorphous sample, which should have gtg conformations. Therefore, we propose that the band at 856 cm^–1^, which appears in the semicrystalline sample, is mainly attributed to the ω(C–H)_ring_ forming hydrogen bonds.

Finally, the theoretical spectra of isolated molecular conformers in the spectral region where the ν(C=O) is present (Figure 11b,e) appear blue-shifted in comparison to the experimental ones. Nevertheless, the calculated spectra for the crystalline models (Figure 11h) are red-shifted in comparison to those of the isolated conformers and match rather well the experimental features. Once more, the red shift is greater for the *syn–syn* conformations than for the *syn–anti* ones, further corroborating the importance of hydrogen bonding. In this region, the experimentally observed present several contributions both before and after the crystallization, attributed to *syn* and *anti* conformations of the C=O groups in the FDCA moiety.^9^ The predicted vibrational frequencies of the isolated molecular conformers with *syn–syn* conformations appear at higher wavenumbers where experimentally very low adsorption is observed. This suggests that experimentally the carbonyl groups in this kind of conformations are forming hydrogen bonds. In the case of the calculated spectra of crystalline models, our results show that *anti* and *syn* conformations could match with the observed bands. However, the predicted spectra for *syn–anti* conformers, structures (d) and (e), show a major contribution at low frequencies, which is due to the *anti* C=O. Meanwhile, the experimental results reveal that the low-frequency peak of the ν(C=O) band is blue-shifted during the crystallization. This feature could indicate a slight reduction in the number of *anti* configurations in favor of *syn* conformers in the crystallization process.

## 4 Conclusions

Here, we have demonstrated the potential use of FTIR spectroscopy to monitor in real-time hydrogen bonding during polymer crystallization. The FTIR real-time experiments performed *in situ* during the isothermal crystallization of poly(trimethylene 2,5-furandicarboxylate) (PTF) reveal the evolution of hydrogen bonding with crystallization time, while GIWAXS provides information about crystal formation. By using density functional theory (DFT) to perform *ab initio* calculations, the FTIR spectra for different theoretical structures were simulated. By comparing experiments and theory, a precise band assignment and its evolution with crystallization time can be obtained. On the basis of DFT calculations and from the comparison with both FTIR and GIWAXS experiments, for the first time, a proposal for the crystalline structure of poly(trimethylene 2,5-furandicarboxylate) was discussed. Our results demonstrate that hydrogen bonding is present in both the crystalline and the amorphous phases, and its rearrangement can be considered as a significant driving force for crystallization of poly(alkylene 2,5-furanoate)s.

## Supporting Information Available

FTIR spectra deconvolution procedure and about the density functional theory (DFT) ab initio calculations. The DFT modeling for a model of an infinite PTF chain (1D model chain) is also included as well as the X-ray simulated patterns for all of the crystalline structures investigated (PDF)
Crystallographic data (CIF)

## Supplementary Material

ma3c02471_si_001.pdf

ma3c02471_si_002.cif
