# Supplementary material for: Study of the Crystal Structure and Hydrogen Bonding during Cold Crystallization of Poly(trimethylene 2,5-furandicarboxylate)
Source: Macromolecules. 2024 Feb 26;57(5):2218–29. doi: 10.1021/acs.macromol.3c02471 (PMC10938886; doi:10.1021/acs.macromol.3c02471)
Supplement: Supplementary file 1 — ma3c02471_si_001.pdf [file ma3c02471_si_001.pdf]

SUPPLEMENTARY INFORMATION FILE

Study of the crystal structure and hydrogen bonding during cold crystallization of Poly(trimethylene 2,5-furandicarboxylate)

Oscar Toledano[1,3], Oscar Gálvez[2,+,*], Mikel Sanz[2,+], Carlos G. Arcos[2+], Esther Rebollar[4,+], Aurora Nogales[3+,], Mari Cruz García-Gutiérrez[3,+], Gonzalo Santoro[3,+], Izabela Irska[5], Sandra Paszkiewicz[5], Anna Szymczyk[5], Tiberio A. Ezquerra[3,+,*].*

[1]CICECO – Aveiro Institute of Materials, Universidade de Aveiro, Aveiro, Portugal.

[2]Universidad Nacional de Educación a Distancia (UNED), Depto. Física Interdisciplinar, Fac. Ciencias Av. de Esparta s/n, 28232, Las Rozas, Madrid, Spain.

[3]Instituto de Estructura de la Materia, IEM-CSIC, Serrano 121, 28006 Madrid, Spain.

[4]Instituto de Química Física Blas Cabrera, IQF-CSIC, Serrano 119, 28006 Madrid, Spain.

[5]Department of Mechanical Engineering and Mechatronics, West Pomeranian University of Technology, Al. Piastów 19, PL 70310 Szczecin, Poland.

[+] NANOesMAT, UNED, Unidad Asociada al CSIC por el IEM y el IQF, Av. de Esparta s/n, 28232, Las Rozas, Madrid, Spain.

A1: Spectra deconvolution

Selected bands of the infrared spectra represented in Figure 6 of the main text, that were registered during the crystallization process, have been deconvoluted in order to estimate the contribution of the peaks associated to the crystalline phase to the shape and area of the whole band. To achieve this goal, firstly, some representative spectral regions have been selected: 800-750 cm^{-1}, 880-800 cm^{-1}, 1005-950 cm^{-1}, and 1605-1563 cm^{-1}, which shows relevant changes during crystallization, as mentioned in the main text of the article (Figure 6). For each range, baseline correction has been performed for a better deconvolution of the bands.

The bands in the spectral region of 800-750 cm^{-1}, 880-800 cm^{-1} and 1005-950 cm^{-1}show similar features, shape and area, at both room temperature and the beginning of the cold crystallization at 80 ºC (Figure SF1a and SF1b). During crystallization, new bands emerge, associated with the crystal structure (Figure SF1c and SF2c). The fraction of the emerging contributions to the global area of the band can be estimated for each spectra step (see Figure 6 of the main article).

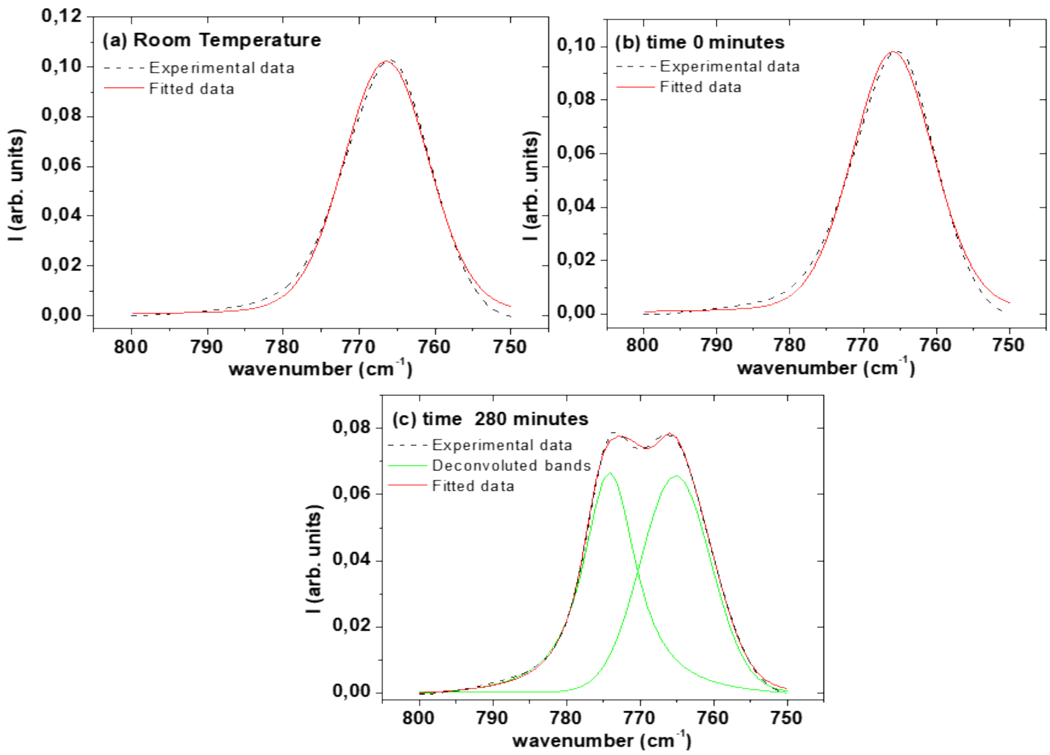

Figure SF1: Experimental FTIR spectrum band (dash line) in the range of 800-750 cm^{-1} with deconvoluted bands (green) and fitted data (red) at a) Room Temperature and heated at 80 ºC for b) 0 minutes and c) 280 minutes.

In the case of the spectral region at 1605-1563 cm^{-1} (stretching C=C bonds of the furane ring), a small peak around 1574 cm^{-1}, that could be associated to the presence of syn-syn conformations (according to our calculations), is already present at room temperature (see Figure SF2a). The percentage of the area of this peak at room temperature is around 12 %. This peak grows and narrows when the crystallization temperature is reached. The initial contribution of this peak to the global area at 80 ºC of around 17 % (see Figure SF2b). To take into account only the contribution of the peak associated with the crystalline phase, the area of this peak at room temperature has been subtracted to that obtained at different times of the crystallization. Figure SF2c also shows the deconvolution of the FTIR spectra of the sample at the final step of the crystallization process.

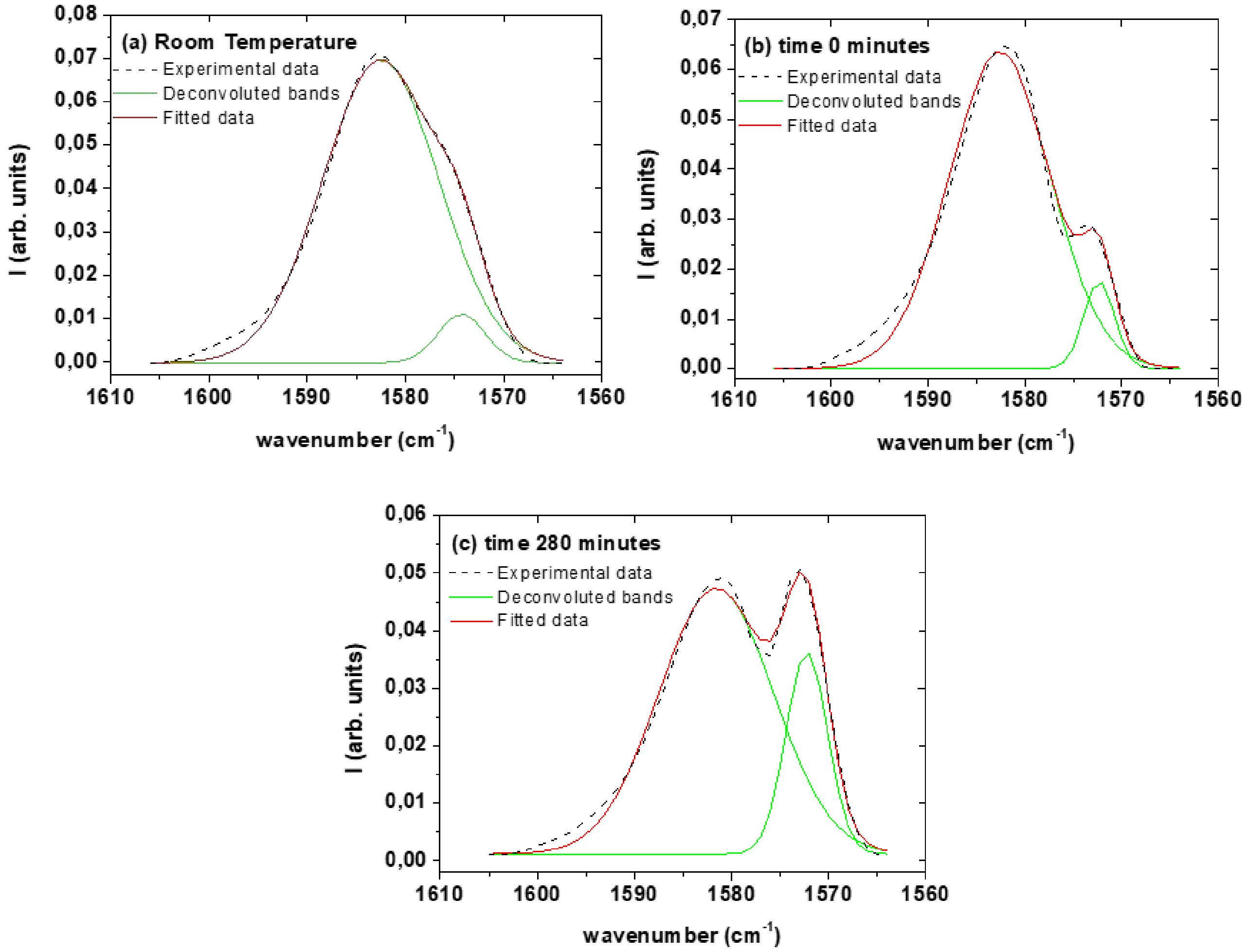

Figure SF2: Experimental FTIR spectrum band (dash line) in the range of 1605-1563 cm^{-1} with deconvoluted bands (green) and fitted data (red) at a) Room Temperature and heated at 80 ºC for b) 0 minutes and c) 280 minutes.

A2: Density Functional Theory *ab initio* calculations

In order to evaluate the stability of the different conformers of the PTF monomer, we have performed DFT calculations on three different types of systems. i) An isolated PTF model monomer, composed of two 1,3-propanediol (PDO) and one 2,5-furandicarboxylic acid (FDCA) units, PDO_2FDCA_1. ii) A model of an infinite PTF chain (1D model chain) consisting of an isolated thread with periodic boundary conditions in the direction of the thread. These type of models have been used for poly(butylene terephthalate) (PBT)[2]. iii) A bulk crystalline structure with periodic boundary conditions in all directions. Here, we will present the results obtained for the model molecular monomer and for the isolated thread that were omitted in the main text. In addition, the calculated diffraction patterns of the proposed crystalline structures (see Figure SF5) are shown and compared with the experimental ones.

A2.1 Isolated PTF model monomer

In the case of the model molecular monomer, nine different conformers were selected as representative cases (see Figure 10 in the main text) because the number of possible structures is too large to be carried out (see discussion below). The structural variables that can be varied without significant energy increments on the model molecular monomer are mainly the dihedral angles that determine the positions of the carbonyl atoms, which can be either in *syn* or *anti* configuration, and the dihedral angles along the alkyl chain, which could be in a *trans* or *gauche* configuration. Considering that in the model molecular monomer, there are two carbonyl groups and six significant dihedral angles that determine the alkyl chains orientations, 256 possible configurations can be formed. This number grows to 2916 if one aims to distinguish the two different orientations for the *gauche* defects in the alkyl chain. Despite some of these configurations will be equivalent due to symmetric considerations, the configurational space is still considerable to be fully explored. In the case of the carbonyl groups orientations, only three possible non-equivalent configurations can be formed: the *syn-syn*, *anti-anti*, and *syn-anti*, labelled with “ss”, “aa”, and “sa”, respectively. In the

case of the alkyl chains, we have studied three representative cases, where both alkyl chains have identical sequence of *trans* (t) and *gauche* (g) ordering (as moving apart from the furan ring). In the first of them, the extended *all-trans* structure is explored (first column in Fig. 10). In the second and third cases, two combinations of *gauche* and *trans* configurations were proposed, labelled as "gtg" and "tgt" (second and third column, respectively). The calculated infrared absorption spectra of the configurations shown in Figure 10 of the main text are depicted in Figure SF3, along with the experimental spectra obtained before and after the crystallization process.

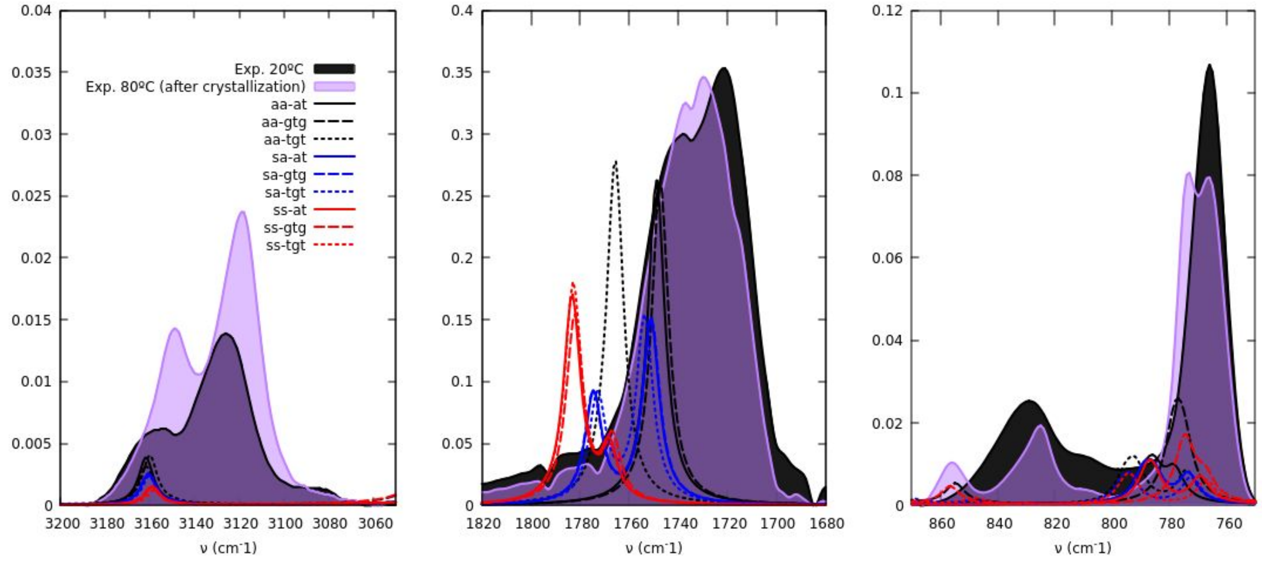

Figure SF3: Experimental FTIR spectra before (dark purple) and after the crystallization process (light purple), along with the calculated spectra of the structures shown in Figure 10.

A2.2 1D model PTF chain

When the model PTF chain is formed, the thread is arranged in a 1D periodic structure, which will entail some additional constraints to the molecular configurations explored previously. In Figure SF4, we can see five of this kind of 1D configurations and their relative energies per PTF monomer unit. In this case, the *anti-anti* configuration, which was the most stable for the molecular structure, now yields the highest relative energy values. This can be explained due to the more extended structures. The *syn-syn* or *syn-anti* configurations lead to a better match with the 1D periodic boundary

conditions than the *anti-anti* ones. This fact reduces the stresses and lead to more stable structures. In fact, *anti-anti* configurations of 1D threats generate more coiled structures. We can see in Figure SF4 that for *syn-syn* conformations adjacent furan rings tend to be equally oriented, while in the *syn-anti* structures appear with alternated orientations, due to the odd number of methylene groups present in the PTF alkyl chain. This will lead to longer unit cells in the *syn-anti* configurations, which comprise two PTF monomer units instead of the unit cell predicted for the *syn-syn* conformers, which only include a single monomer. Thus, the stresses induced by the formation of periodic conditions will be shared by the two monomers included in the unit cell of *syn-anti* conformations enabling the formation of more stable structures, as it was effectively observed. As mentioned in the main text, in the bulk crystalline phases this trend is reversed and the *syn-syn* configurations become more stable due to the interchain hydrogen bond interactions formed between adjacent threads, which are stronger in the *syn-syn* configurations than in the *syn-anti*.

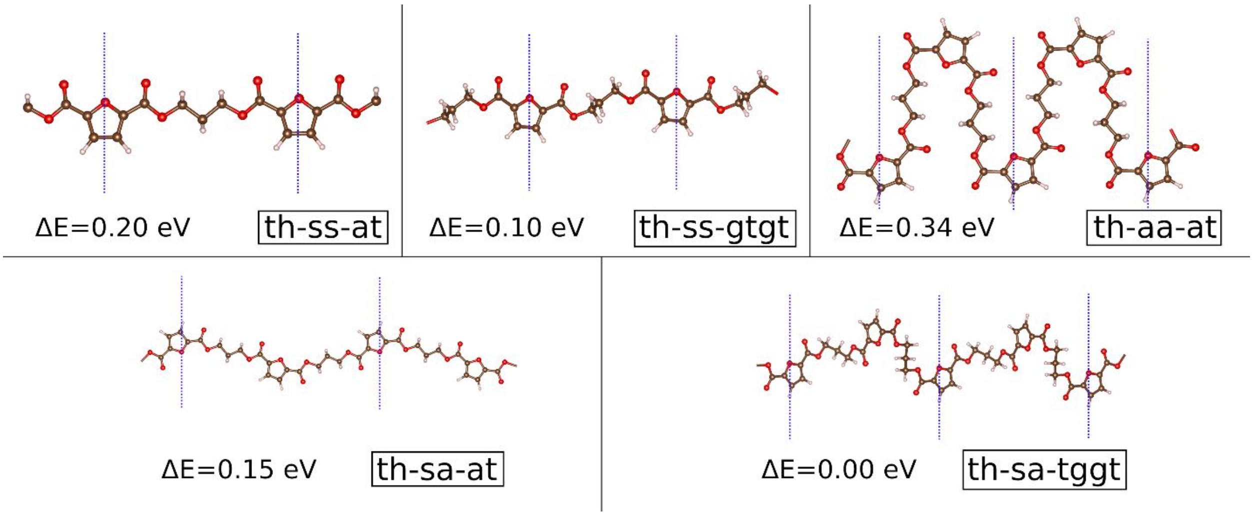

Figure SF4: Periodic PTF thread configurations. The relative energy per unit cell is displayed for the periodic cells. Blue dotted lines mark the 1D unit cell limits.

The calculated infrared absorption spectra of the 1D configurations shown in Figure SF4 are depicted in Figure SF5, along with the experimental spectra obtained before and after the crystallization process. As mentioned in the main text, it can be noticed that some of the bands cannot be explained without the formation of the interchain hydrogen bonds. Particularly, the $(C\text{-}H)_{ring}$

stretching occurring at low frequencies, around 3120 cm^{-1}, the C=O stretching, centered at approximately 1720 cm^{-1}, and the $(C\text{-}H)_{ring}$ wagging, at around 825 cm^{-1}, can only be observed when interchain hydrogen bonds are formed between nearby polymer chains.

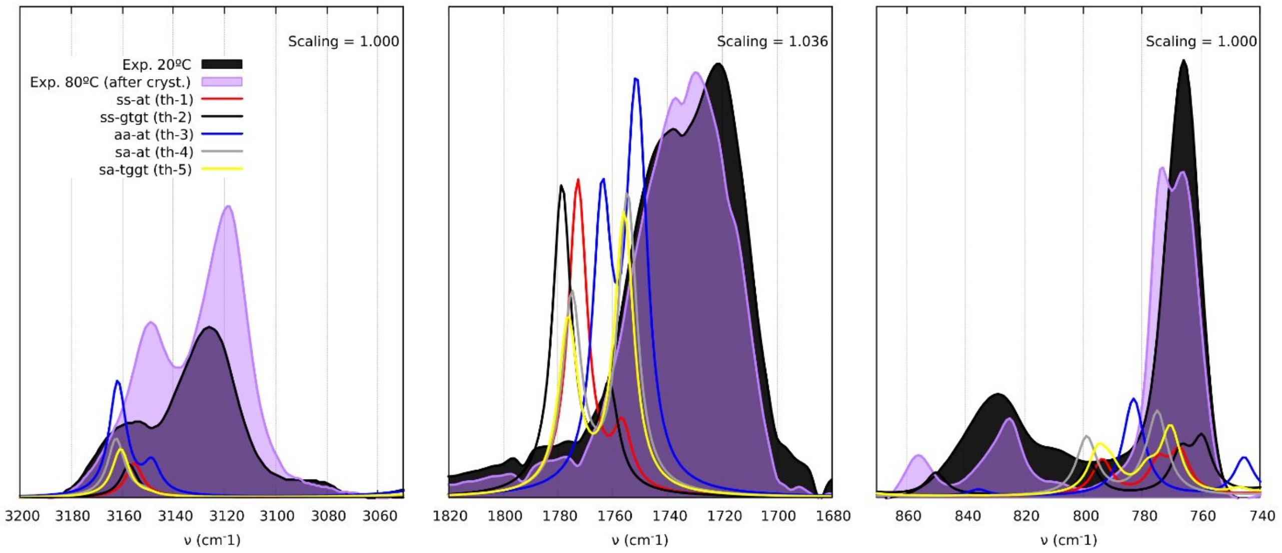

Figure SF5: Experimental FTIR spectra before (dark purple) and after the crystallization process (light purple), along with the calculated spectra of the structures shown in Figure SF4.

A2.3 Bulk crystalline structures

Figure 8, in the main text, shows five probable crystalline structures selected to be candidates for that of PTF. Figure SF6 shows the simulated diffraction patterns of the crystalline structures proposed. In addition, we have included in Figure SF6: an experimental pattern of a well cold crystallized PTF sample (T_c= 160 ^oC, t= 6 hours, Figure 2) and another diffractogram from the literature corresponding to a PTF crystallized from the melt[1]. The simulated diffractograms of the structures (a), (b), (d) and (e) lead to diffraction patterns that do not match the experimental ones. On the one hand, in different cases, some of the main observed peaks are absent (for structures (a) and (b)), or strong peaks are predicted but not observed (in structures (b), (d), and (e)). On the other hand, the agreement of the diffraction pattern of the expanded structure (c) with those observed experimentally is significant both in peak positions as well as in relative intensities.

As mentioned in the main text, the unit cell parameters of some of the structures proposed were expanded from those obtained after geometrical relaxation, in order to achieve a better fitting of the peak positions of the diffraction patterns with those obtained experimentally. In this process, a systematic variation of the unit cell parameters, lengths and angles, was carried out. Configurations that showed the smallest deviation of the peak positions with those obtained experimentally were selected. The maximum deviations allowed in this process from the structural parameters obtained after full relaxation of the structure were 0.5 Å and 20º for length and angles, respectively. In addition, configurations with densities that differ more than 0.05 g/cm^3 from the one measured in a semicrystalline PTF sample[3], ρ=1.426 g/cm^3, were disregarded. The density of the crystalline structure was inferred from the amorphous ρ=1.355 g/cm^3 and semicrystalline ρ=1.377 g/cm^3 density values, and the crystallinity of the sample, which was estimated to be $\chi_c = 0.31$. In the case of structures (b) and (d), this process was not performed, due to their predicted diffraction pattern reveal a lack of the experimental peak at q=7.25 nm^{-1} (see below). In these cases, only the diffraction patterns of the relaxed unit cells without distortion are shown in Figure SF6. In first place, we can observe that the experimental diffractograms show a peak at q=7.25 nm^{-1}, which can only be assigned to the (0,0,1) plane. Only structures (a), (c) and (e) show a diffraction peak in this region, so we will only consider these structures for a further refinement of the unit cell, as described previously. Regarding the (e) structure, we can see that even if some of the diffraction peaks are close to the experimental ones, there is an extra peak at q=3.73 nm^{-1} which is not observed experimentally, and which corresponds to the (0,0,1) diffraction plane of the simulated structure. Thus, this structure seems unlikely to participate in the crystalline phase of PTF. The diffraction pattern of structure (a), on the other hand, shows a nice agreement with the experimental ones for some of the peaks. However, the diffraction peak experimentally observed around q=13.4 nm^{-1} is not obtained for this structure. In addition, the relative intensities of the peaks at 10.5 nm^{-1} and 1.7 nm^{-1} do not match with those observed experimentally. Finally, we can see that the agreement of the diffraction pattern of structure (c) with the experimental ones is remarkable. The major deviation is obtained for the peak at q=13.4

nm^{-1}, for only 0.4 nm^{-1} (the predicted peak is situated at q=13.8 nm^{-1}). The positions of the rest of the peaks and their relative intensities are in nice agreement with the experiments.

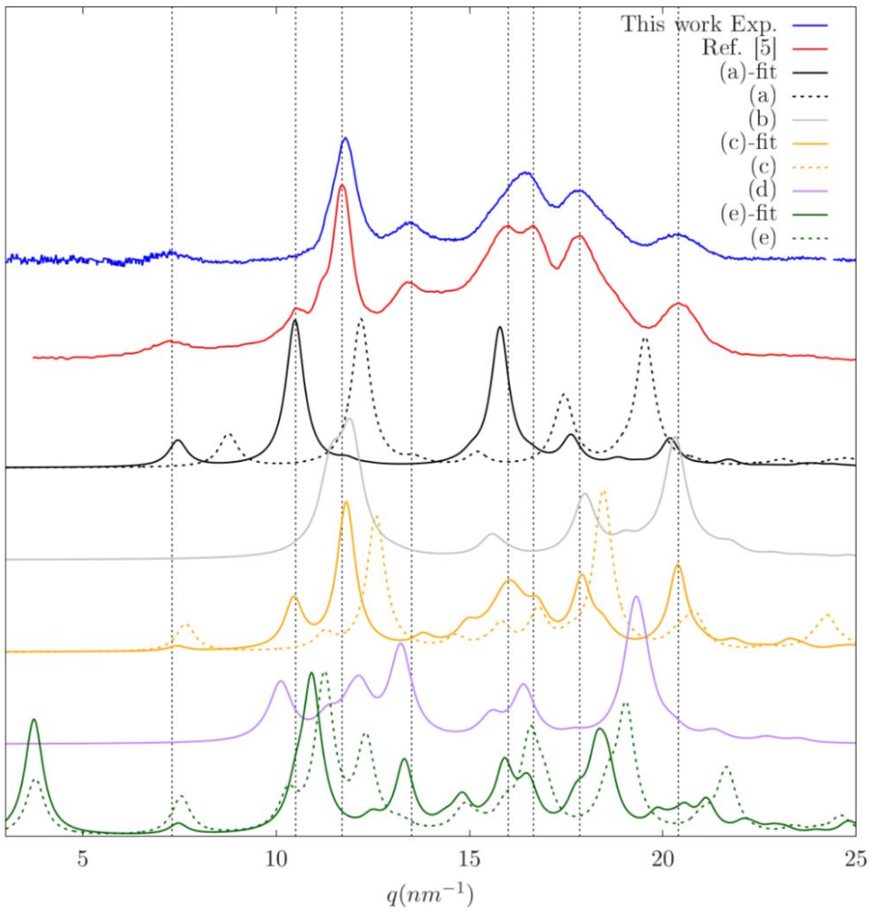

Figure SF6: Calculated powder diffraction patterns of the crystalline structures shown in Figure 8, along with the experimental patterns obtained in this work after crystallization and in reference[1]. Vertical dashed lines are placed at the main diffraction peak positions observed experimentally as a guide for the eye.

References

1. Papageorgiou, G. Z.; Papageorgiou, D. G.; Tsanaktsis, V.; Bikiaris, D. N. Synthesis of the bio-based polyester poly(propylene 2,5-furan dicarboxylate). Comparison of thermal behavior and solid state structure with its terephthalate and naphthalate homologues. *Polymer* **2015,** 62, 28-38 DOI: 10.1016/j.polymer.2015.01.080.
2. Milani, A.; Galimberti, D. Polymorphism of Poly(butylene terephthalate) Investigated by Means of Periodic Density Functional Theory Calculations. *Macromolecules* **2014,** 47 (3), 1046-1052 DOI: 10.1021/ma402602f.
3. Vannini, M.; Marchese, P.; Celli, A.; Lorenzetti, C. Fully biobased poly(propylene 2,5-furandicarboxylate) for packaging applications: excellent barrier properties as a function of crystallinity. *Green Chemistry* **2015,** 17 (8), 4162-4166 DOI: 10.1039/c5gc00991j.
